# Temporal brain transcriptome analysis reveals key pathological events after germinal matrix hemorrhage in neonatal rats

**DOI:** 10.1177/0271678X221098811

**Published:** 2022-05-01

**Authors:** Juan Song, Gisela Nilsson, Yiran Xu, Aura Zelco, Eridan Rocha-Ferreira, Yafeng Wang, Xiaoli Zhang, Shan Zhang, Joakim Ek, Henrik Hagberg, Changlian Zhu, Xiaoyang Wang

**Affiliations:** 1 Centre for Perinatal Medicine and Health, Institute of Neuroscience and Physiology, Sahlgrenska Academy, University of Gothenburg, Gothenburg, Sweden; 2 Henan Key Laboratory of Child Brain Injury and Henan Pediatric Clinical Research Center, Third Affiliated Hospital and Institute of Neuroscience of Zhengzhou University, Zhengzhou, China; 3 Centre for Perinatal Medicine and Health, Institute of Clinical Sciences, University of Gothenburg, Gothenburg, Sweden; 4 Henan Provincial Key Laboratory of Children’s Genetics and Metabolic Diseases, Children’s Hospital Affiliated to Zhengzhou University, Zhengzhou, China; 5 Center for Brain Repair and Rehabilitation, Institute of Neuroscience and Physiology, Sahlgrenska Academy, University of Gothenburg, Sweden

**Keywords:** Germinal matrix hemorrhage, RNA-sequencing, mitochondria, ferroptosis, neurodevelopment

## Abstract

Germinal matrix hemorrhage (GMH) is a common complication in preterm infants and is associated with high risk of adverse neurodevelopmental outcomes. We used a rat GMH model and performed RNA sequencing to investigate the signaling pathways and biological processes following hemorrhage. GMH induced brain injury characterized by early hematoma and subsequent tissue loss. At 6 hours after GMH, gene expression indicated an increase in mitochondrial activity such as ATP metabolism and oxidative phosphorylation along with upregulation of cytoprotective pathways and heme metabolism. At 24 hours after GMH, the expression pattern suggested an increase in cell cycle progression and downregulation of neurodevelopmental-related pathways. At 72 hours after GMH, there was an increase in genes related to inflammation and an upregulation of ferroptosis. Hemoglobin components and genes related to heme metabolism and ferroptosis such as *Hmox1, Alox15*, and *Alas2* were among the most upregulated genes. We observed dysregulation of processes involved in development, mitochondrial function, cholesterol biosynthesis, and inflammation, all of which contribute to neurodevelopmental deterioration following GMH. This study is the first temporal transcriptome profile providing a comprehensive overview of the molecular mechanisms underlying brain injury following GMH, and it provides useful guidance in the search for therapeutic interventions.

## Introduction

Germinal matrix hemorrhage (GMH), including periventricular/intraventricular hemorrhage, is one of the most common complications and is a major cause of brain injury in preterm infants and is associated with increased mortality, especially in those born before 32 weeks of gestation or with a birth weight <1500 g.^1–3^

The germinal matrix, present in the brain between 8 and 36 weeks of gestation in humans, is a specialized area where neuronal and glial cell differentiation takes place. The rich vascular network of the germinal matrix is thin and fragile, and the immature brain lacks adequate autoregulation of the cerebral blood flow; therefore, fluctuations in blood flow can result in the rupture of blood vessels and subsequent hemorrhage. The hemorrhage is sometimes restricted to the germinal matrix, but more often it extends into the lateral ventricle and in severe cases into the parenchyma.^4,5^ GMH is divided into four grades, and about 5–8% of preterm infants with a gestational age of 22–32 weeks develop severe GMH (grade III or IV).^6–8^ GMH can lead to adverse neurodevelopmental outcomes such as cerebral palsy, cognitive deficits, behavioral disorders, or a combination of these sequelae.^3,9–12^

Brain injury after GMH consists of two different phases – the primary injury and the secondary injury. The primary brain injuries are mostly caused by a mass effect induced by the hematoma, including increased intracranial pressure and blockage of cerebrospinal fluid.^13^ The secondary brain injury is caused by hemoglobin degradation products, inflammation, death of neuron and glia cells, arrest of preoligodendrocyte maturation, and microglia activation, all of which might lead to post-hemorrhagic ventricular dilatation, periventricular leukomalacia, or diffuse white matter injury.^14–16^

There are currently no specific therapies to prevent GMH or to treat the adverse outcomes resulting from the insult. Thus, studies focusing on potential mechanisms of GMH in preterm infants are important in order to devise new therapies. Rodent models of neonatal GMH have been shown to be helpful to investigate mechanisms of GMH and subsequent brain injury development. However, we lack an overall understanding of the biological processes, including gene expression patterns and signaling pathways, involved in the pathogenesis of GMH and in the development of secondary brain injury.

In this study, a model of preterm GMH in postnatal day (P) 5 rats was used, and RNA sequencing (RNA-seq) was performed at different time points after insult to identify changes in the transcription profile in the brain after GMH. The goal was to provide an overall picture and a deeper understanding of the mechanisms of brain injury caused by GMH in order to support the development of effective therapeutic targets for this debilitating condition.

## Materials and methods

### Animals

Wistar rats of both sexes were used in the experiments. Day of birth was defined as P0. Animals were bred and housed at Experimental Biomedicine, University of Gothenburg, with water and food available ad libitum. Animal experiments conformed to guidelines established by the Swedish Board of Agriculture (SJVFS 2015: 38) and were approved by the Gothenburg Animal Ethics Committee (ethical number 2042/18) and reported in accordance with the ARRIVE guidelines (Animal Research: Reporting In Vivo Experiments).^17^

### GMH induction

**Figure 1.**
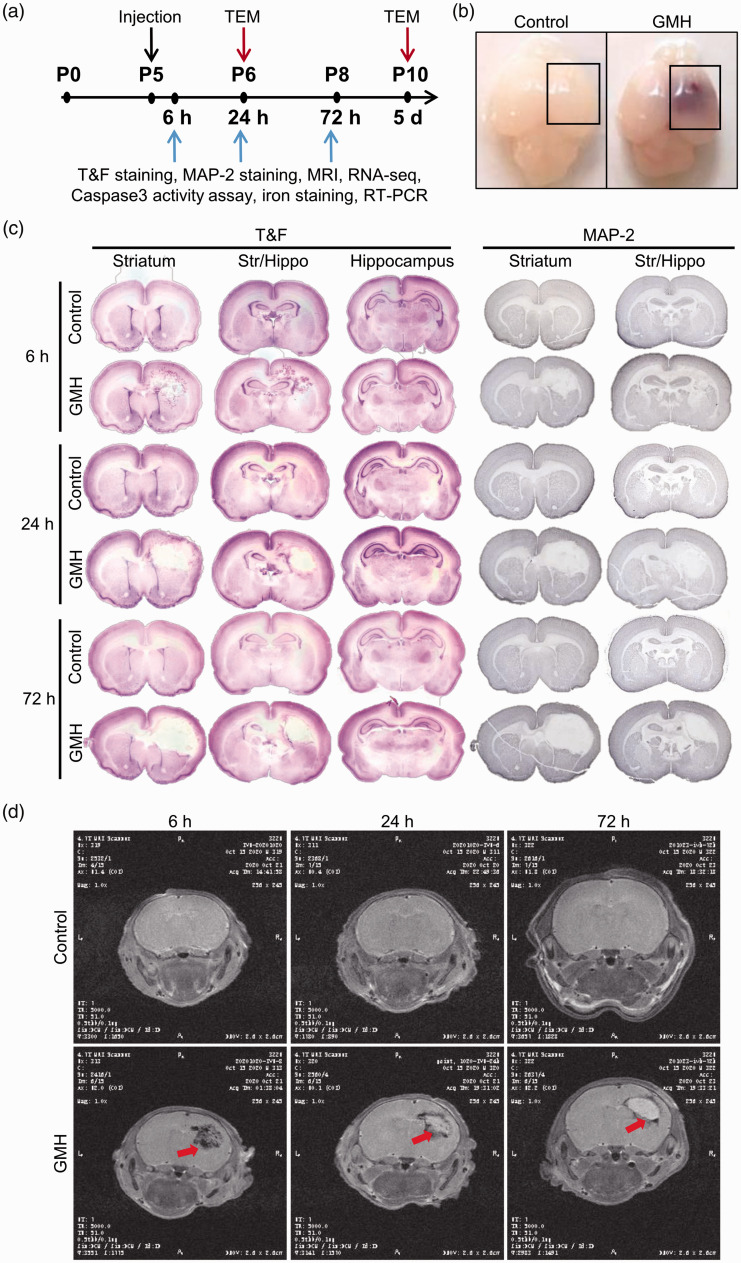
Experimental design and the GMH model in P5 rats. (a) Schematic presentation of the experimental design. (b) Photographs of rat brains at 6 hours after intracranial injection with saline (control) or collagenase (GMH). The mark shows the part of the brain used for RNA and protein preparations. (c) Photomicrographs of coronal brain sections at the level of the striatum, at the intermediate level between the striatum and hippocampus (Str/Hippo), and at the hippocampus level stained with thionin and fuchsin or anti-MAP-2 antibody at 6 hours, 24 hours, and 72 hours after intracranial injection of collagenase or saline and (d) MRI T2WI images of rat brains at 6 hours, 24 hours, and 72 hours after intracranial injection of collagenase or saline.

At P5, rat pups were randomly allocated into control and GMH groups. Following anesthesia with isoflurane (5% for induction and 3.5% for maintenance) in a mixture of oxygen and nitrogen, rats were injected in the right striatum with either collagenase VII (0.3 U, 1000–3000 CDU/mg solid, C2399, Sigma-Aldrich, Saint Louis, USA) to induce GMH or with saline as control. Injections were administered at 1 μL/min for 2 min in the right hemisphere 1 mm rostral of the bregma and 4 mm lateral of the midline, and 3.5 mm in depth, using a 27 G (0.4 mm) needle and a 1 mL Hamilton syringe connected to an infusion pump (CMA/100 microinjection pump) as previously described.^18^ The pups were allowed to recover on a heating pad at 37°C after completing the procedure and then were returned to their dams until being sacrificed at different time points according to the experimental design (Figure 1(a)). The duration of the procedure was less than 5 min/animal.

### Thionin and fuchsin staining

Rat pups were deeply anesthetized with isoflurane and intracardially perfused with cold saline followed by cold Histofix (Histolab, Sweden), and their brains were dissected out and post-fixed in Histofix. Following dehydration and paraffin embedding, the brains were cut with a microtome into 7 µm thick coronal sections. Brain sections were deparaffinated in xylene and rehydrated in graded alcohol of decreasing concentrations and distilled H_2_O followed by staining with thionin for 4 min and acid fuchsin for 2 min. This was followed by dehydration and mounting using Pertex mounting medium (Histolab, Sweden).

### Immunohistochemistry staining

After deparaffination in xylene and rehydration in an alcohol gradient, brain sections were boiled in 0.01 M citric acid buffer (pH 6.0) followed by blocking with 3% H_2_O_2_ and 1% horse serum. Sections were incubated with anti-microtubule associated protein 2 (MAP-2) antibody (1:1000 dilution, M4403, Sigma-Aldrich) followed by horse anti-mouse secondary antibody. After incubation with ABC elite and submerging into 0.5 mg/ml 3, 3′-diaminobenzidine (DAB), enhanced with NiSO_4_, sections were dehydrated and mounted with Pertex (Histolab, Sweden). Images were taken using a light microscope (Olympus BX60, Japan).

### Magnetic resonance imaging

T2-weighted imaging (T2WI) was performed with a small animal magnetic resonance imaging (MRI) device (MR Solutions 4.7 T) at 6 hours, 24 hours, and 72 hours after intracranial injection with collagenase VII to induce GMH or with saline as controls (n = 5/group). During the entire MRI scan, the rats were anesthetized with isoflurane via a gas anesthesia system (5% for induction and 2% for maintenance). T2WI used the fast spin echo sequence, and scan parameters were set as follows: repetition time =5000 ms, echo time = 51 ms, reverse angle = 180°, field of view = 25 mm × 25 mm, matrix = 256 × 256. The coronal T2WI was performed with the number of layers = 20 and thickness = 0.5 mm. MRI images were collected using Preclinical Scan 1.2 software.

### RNA-seq and analysis

In total, 60 replicates were used for RNA-seq, consisting of 10 replicates (5 males and 5 females) per timepoint and treatment. Each replicate contained a sample from one animal. The number of replicates in each group was decided according to the recommendations.^19^ Each timepoint had an group of age-matched saline-injected rats as controls. Rat pups were intracardially perfused with cold saline. Ipsilateral hemispheres without the front and back parts of the brains were collected (Figure 1(b)). After quick manual homogenization in RNase-free PBS, total RNA was extracted using an miRNeasy Mini kit (Qiagen, Hilden, Germany) and quantified using a spectrophotometer (NanoDrop 2000, Thermo Scientific) at 260 nm absorbance. RNA was sent for sequencing to Novogene, UK. In brief, quality control of sample purity and integrity was performed using a Nanodrop and Agilent 2100 and the libraries were sequenced on an Illumina NovaSeq platform to generate 150 bp paired-end reads according to the manufacturer’s instructions. Mapping to the reference genome was performed using HISAT2 software (2.0), and quantification was performed using HTseq software (0.6.1). The transcriptome sequencing data generated in this study are available from the BioSample database (BioProject ID: PRJNA756842).

Differential gene expression analysis was performed using DEseq2 software (1.20.0), and the resulting *p*-values were adjusted using the Benjamini and Hochberg approach for controlling the false discovery rate. Genes with an adjusted *p*-value <0.05 according to DESeq2 were assigned as differentially expressed genes (DEGs). The enrichment analysis of DEGs, including Gene Ontology (GO) enrichment analysis, was performed using gProfiler (https://biit.cs.ut.ee/gprofiler/)^20^ and GOplot (1.0.2).^21^ GO terms with corrected *p*-values less than 0.05 were considered to be significantly enriched by DEGs. The most significant terms are displayed with the z-score calculated by GOplot. Kyoto Encyclopedia of Genes and Genomes (KEGG) pathway and Gene Set Enrichment Analysis (GSEA) was implemented with the clusterProfiler software (4.0.5)^22,23^ (http://www.genome.jp/kegg/). Regulated molecular pathways were explored by analyzing the significant DEGs using Ingenuity Pathway Analysis (70750971) (IPA, Qiagen) with a cut-off adjusted *p*-value of <0.05.

Immune cell infiltration analysis was performed using R software (https://www.r-project.org/) with the MCPcounter (1.1)^24^ and XCELL (1.1.0)^25^ packages. The normalized gene expression using log2 (FPKM + 1) was used as input data to analyze the distribution levels of CD8^+^/CD4^+^ T-cells, macrophages, neutrophils, myeloid dendritic cells, monocytes, NK cells, and B-cells. Figures were generated with the ggplot2 (3.3.5) and Pheatmap R packages (1.0.12) or Qlucore Software (Lund, Sweden).

### Caspase-3 activity assay

At 6 hours, 24 hours, and 72 hours after GMH induction, rat pups (n = 10/group) were intracardially perfused with cold saline. The sample size was decided based on our previous experiments.^26–28^ After removing the front and back parts of the brain (Figure 1(b)), brain hemispheres ipsilateral to the hemorrhage (approximately 200 mg) were collected. Tissues were placed in ice-cold RNase-free PBS including protease inhibitors and phosphatase inhibitors and manually homogenized using a standard tissue grinder and sonicated for 5–10 s. Following centrifugation at 10,000 × *g* at 4°C for 10 min, the supernatant was used for fluorometric assay of caspase-3-like activity, as described previously.^29^ In brief, 20 µL of each sample was added to a microplate and mixed with 80 µL extraction buffer (dimethylamino propane sulphonic acid, 1% protease inhibitor cocktail (P8340; Sigma), and 1 mM phenylmethylsulfonylfluoride). After incubation for 15 min at room temperature, 100 µL assay buffer (50 mM Tris, 100 mM NaCl, 5 mM EDTA, 1 mM EGTA, 4 mM DTT, and 1 mM PMSF) containing 25 µM caspase-3 substrate (Ac-Asp-Glu-Val-Asp-aminomethyl coumarin (AMC; #SAP3171v, Peptide Int.)) was added. Cleavage of the substrate was measured at 37°C using a Spectramax Gemini microplate fluorometer (Molecular Devices, Sunnyvale, CA) with an excitation wavelength of 380 nm and emission wavelength of 460 nm. The kinetics were followed at 2 min intervals for 1 h, and Vmax was calculated from the entire linear part of the curve. Standard points with AMC in the appropriate buffer were used to express the data in picomoles of AMC (7-amino-4-methyl-coumarin) formed per minute and per milligram of protein.

### Iron staining

DAB-enhanced Perl’s staining was used to measure iron accumulation after GMH. In brief, brain sections were deparaffinated and incubated in a freshly prepared iron solution (1:1 mixture of 4% potassium ferrocyanide and 1.2 mM hydrochloric acid, Sigma-Aldrich) for 10 min followed by five washes in PBS, incubation in DAB (3, 3′-Diaminobenzidine tetrahydrochloride, Sigma-Aldrich) for 10 minutes, counterstaining with pararosaniline (Sigma-Aldrich), and dehydration and cover-slipping. Slides were imaged, and iron deposition was observed using a light microscope (Olympus BX60, Japan).

### Reverse transcription quantitative PCR

Brain tissue was manually homogenized in RNase-free PBS, and total RNA was extracted using an miRNeasy Mini kit (Cat. No. 205313, Qiagen) and quantified using a spectrophotometer (NanoDrop 2000, Thermo scientific) at 260 nm absorbance (n = 10/group, that was decided based on our previous experiments^30^). A total of 1 μg RNA was used for complementary DNA (cDNA) generation using a QuantiTect Reverse Transcription kit (Qiagen). The reverse transcription protocol included 2 min at 42°C, 30 min at 42°C, and 3 min at 95°C. SYBR Green-based primers (Qiagen) for *Hmox1* (QT00175994), *Hspb1* (QT00179501), and *Alox15* (QT00181265) were used for gene amplification. The QuantiFast SYBR Green (Qiagen) amplification protocol included 45 cycles of 10 s at 95°C and 30 s at 60°C. A Quant-iT OliGreen ssDNA Assay Kit (Thermo Fisher Scientific) was used to determine the amount of single-stranded DNA in each cDNA sample in a fluorometer with excitation at 480 nm and emission at 520 nm. Relative quantification of gene expression was corrected using the cDNA concentration ratio between the samples.

### Transmission electron microscopy (TEM)

Rat pups (n = 5/group, that was decided based on our previous experiments^31^) were intracardially perfused with cold saline followed by cold fixative (0.1 M phosphate buffer pH 7.4, 2.5% glutaraldehyde, and 2% paraformaldehyde). Perihematomal brain tissue samples were prepared using a Leica EM Automatic Microwave Tissue Processor including post-fixation (1% OsO_4_ with 1% K_4_Fe(CN)_6_ followed by 0.5% uranyl acetate in dH_2_O), dehydration, and gradual infiltration of resin at 50°C (Hard Plus Resin 812 kit, EMS). Sections (70–90 nm) were placed on copper slot grids and stained with 2% uranyl acetate and lead citrate. Transmission electron microscope (TEM) images were captured with a Hitachi 7600 TEM in the Microscope Core facility of Peking University.

### Statistical analysis

GraphPad Prism 9.3.1 (GraphPad software, San Diego, CA, USA) was used for statistical analysis of the caspase-3 activity assay data and RT-PCR data. Data are presented as interleaved scatter plots with bars showing the mean with standard deviation. Shapiro-Wilk test and generation of QQ-plots were used to assess data distribution. Data with normal distribution were analyzed using two-way ANOVA and Sidak´s post hoc, and *p* < 0.05 was considered statistically significant.

## Results

### GMH causes secondary brain injury

In this study we used a previously described preterm rat model of GMH in which near-immediate bleeding is caused by collagenase injection in the brain of P5 rat pups.^18,32^ Saline-injected rat pups were used as controls. At 6 hours after collagenase injection, bleeding was visible on the surface of the brain, while no bleeding was observed on the brain of saline-injected control rats (Figure 1(b)). In the brain, bleeding occurred mainly in the striatum of the hemisphere ipsilateral to the injection, with some bleeding expanding into the ventricle and rarely into the contralateral hemisphere. Histochemical staining with thionin and fuchsin and MAP-2 immunohistochemistry staining (Figure 1(c)) showed that GMH caused brain injury in the bleeding area appearing as tissue disruption at 6 hours after collagenase injection, and increasing tissue loss was observed over time. Brain injury was mainly located at the striatum level and frontal hippocampal level, but occasionally at the posterior hippocampal level (Figure 1(c)). This was also observed in the T2WI images, which showed hematoma at 6 hours followed by infarction at 24 and 72 hours post-GMH (Figure 1(d)).

### A large number of genes are differentially expressed in the brain following GMH

To explore the biological processes induced by GMH and the potential molecular mechanisms involved in brain injury development following GMH, we performed RNA-seq of brains collected at 6 hours, 24 hours, and 72 hours after saline or collagenase injection. Unsupervised principal component analysis (PCA) revealed substantial transcriptome differences in gene expression after GMH at all time points examined. In addition, there was a segregation between the groups of different ages (Supplementary Figure 1 A). In total, there were 498, 413, and 1466 DEGs at 6 hours, 24 hours, and 72 hours, respectively, between the control and the GMH groups, with only a few overlapping genes between different time points (Supplementary Figure 1B, Supplementary data 1).

### Genes regulating mitochondria function and cytoprotective pathways are upregulated at 6 hours after GMH

**Figure 2.**
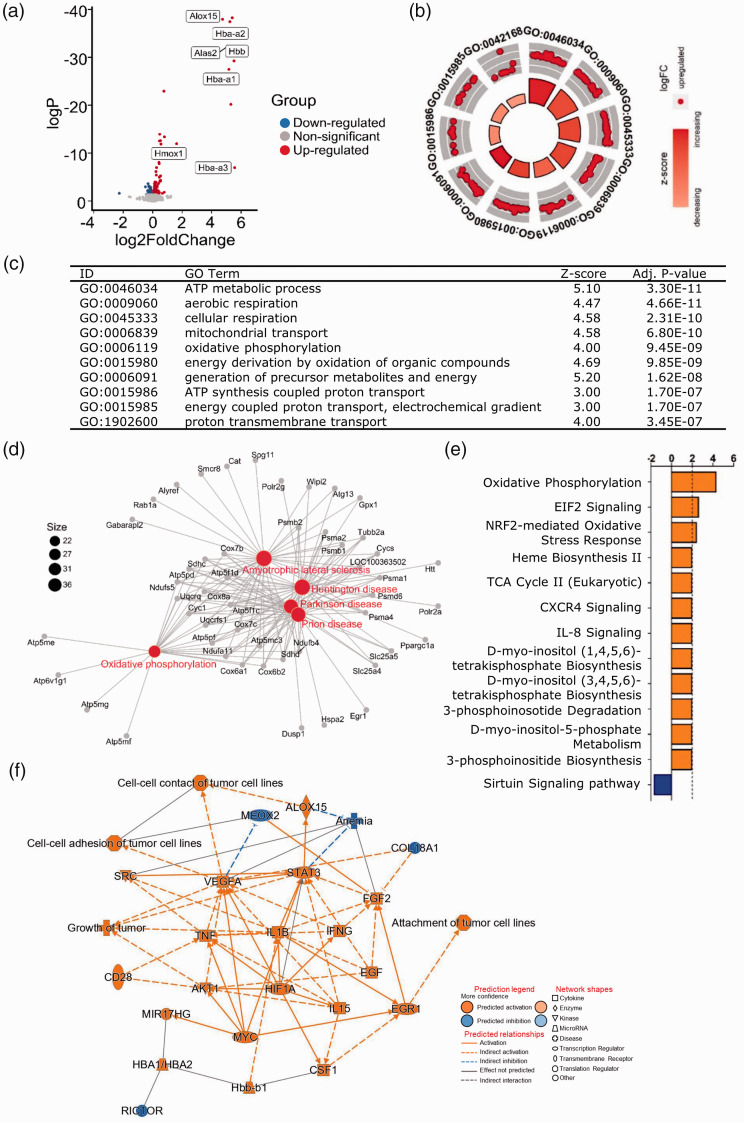
Mitochondrial functions were affected 6 hours after GMH. (a) Volcano plot of DEGs in the GMH group compared to the control group at 6 hours after GMH. *P*-adj <0.001 and |(log2FoldChange)| > 1. (b–c) The most significant GO-enriched biological processes. (d) The most significant KEGG-enriched terms. The canonical pathways (e) and graphical summary of the network (f) predicted by IPA.

At 6 hours after GMH induction, there were 498 DEGs between the control group and the GMH group (Supplementary data 1). Among the top 20 most significantly upregulated genes (Supplementary Table 1) was *Alox15*, a lipoxygenase that catalyzes the generation of various bioactive lipid mediators and whose reaction products have been shown to regulate inflammation and immunity^33^ and to play a role in ferroptosis.^34^ Several of the top 20 significantly upregulated DEGs were hemoglobin components, including *Hbb*, *Hba-a2*, *Hba-a1*, and *Hba-a3*, and genes involved in heme biosynthesis or catabolism, including *Alas2*, *Slc25a39*, *Hmox1*, *Car2*, and *Fech* (Figure 2(a), Supplementary data 1, and Supplementary Table 1). Among the top 20 most significantly downregulated genes were genes playing roles in development, including *Fras1*, *Klj12*, *Rorb*, and *Thsd7a*, and in neuronal functions, such as *Kcnh7*, *Fat3*, *Shc3*, *Tenm4*, *Kcna3*, and *Igsf9*b (Supplementary Table 2).

GOplot analysis showed enrichment in biological processes involved in mitochondrial function and cellular metabolism such as ATP metabolism, cellular respiration, mitochondria transport, oxidative phosphorylation, generation of energy, and formation of precursor metabolites (Figure 2(b) and (c), Supplementary data 2 and 3). In addition, the upregulation of angiogenesis and vasculature development was noted (Supplementary data 2 and 3). KEGG pathway analysis confirmed the upregulation of the oxidative phosphorylation pathway (Figure 2(d)), and this was also predicted to be upregulated by IPA (Figure 2(e)). Furthermore, IPA predicted the upregulation of cytoprotective pathways (e.g., EIF2 signaling and Nfr2-mediated oxidative stress responses) as well as the heme biosynthesis II, TCA cycle II, and inositol metabolism pathways (Figure 2(e) and Supplementary data 4). In addition, transcription factors such as STAT3 and HIF1A as well as growth factors (vascular endothelial growth factor and epidermal growth factor) and hemoglobin components were predicated to be involved in the biological processes affected at 6 hours (Figure 2(f), Supplementary data 5).

Overall, at 6 hours after GMH, the most-affected biological processes were those related to mitochondrial function, cytoprotective pathways and heme metabolism.

### GMH causes gene expression changes representing an increase in cell cycle progression and downregulation of neurodevelopment 24 hours after the insult

**Figure 3. fig3-0271678X221098811:**
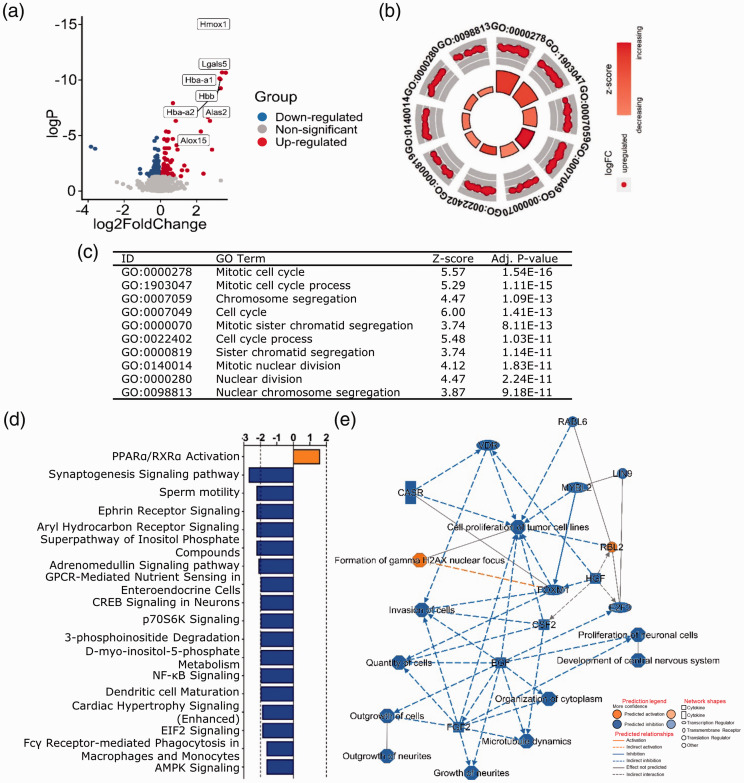
At 24 hours after GMH, cell cycle-related processes were the most significantly enriched. (a) Volcano plot of DEGs at 24 hours after GMH. *P*-adj <0.001 and |(log2FoldChange)| > 1. (b–c) GOplot displaying the most significantly enriched GO biological processes. The canonical pathways (d) and graphical summary of the network (e) predicted by IPA.

At 24 hours after saline or collagenase injection, there were 413 DEGs between the control group and the GMH group. Still at 24 hours, the most significantly upregulated DEGs were genes encoding for hemoglobin components (*Hba-a1*, *Hbb*, and *Hba-a2*) and genes involved in heme biosynthesis and catabolism (*Hmox1* and *Alas2*) (Figure 3(a)). Among the top 20 upregulated genes were also genes regulating the cell cycle, i.e., *Timp1*, *Cdk1*, *Tubb6*, *Pttg1*, *Ccnb1*, *Arhgap11a* and *Cdca8*, and genes involved in cell motility and migration such as *Bin2*, *CD63*, and *Denn2b* (Supplementary data 1, Supplementary Table 3). Cell cycle-regulating genes were also among the top 20 most significantly downregulated genes, i.e., *Hcfc1*, *Ttbk2*, *Mib1*, *Fbxl12*, *Fbxl18*, *Fbxo32, Egr4*, and *Myo5a*, some of which regulate ubiquitination (*Mib1*, *Fbxl12*, *Fbxl18*, and *Fbxo32*) (Supplementary Table 4). In addition, genes having roles in vesicle trafficking, neurotransmission, and neurodevelopment were also among the top 20 most significantly downregulated genes (*Clstn2*, *Prrc2b*, *Mecp2*, *Hip1r*, *Fat3*, *Tenm4*, *Sv2c*, *Tnr*, and *Myo5a*).

GOplot analysis showed enrichment in upregulated cell cycle processes (Figure 3(b) and (c)), wound healing, inflammatory responses, and myeloid cell homeostasis (Supplementary data 2 and 3), while IPA predicted the upregulation of PPARα/RXRα activation and downregulation of multiple pathways, including synaptogenesis, ephrin receptor signaling, aryl hydrocarbon receptor (AhR) signaling, adrenomedullin signaling, CREB signaling in neurons, and the inositol metabolism pathways, all of which are involved in various processes important for CNS development and function^35–39^ (Figure 3(d), Supplementary data 4). The predicted IPA network involved the inhibition of neurite growth, CNS development, and factors regulating cell growth and proliferation, such as epidermal growth factor, fibroblast growth factor, hepatocyte growth factor, and colony stimulating factor 2 (CSF2) (Figure 3(e), Supplementary data 5).

Together these analyses suggested that 24 hours post-GMH the major biological events were characterized by increased cell cycle progression and proliferation and downregulation of neurodevelopmental processes.

### After 72 hours, GMH evokes genes expression related to inflammatory responses and downregulation of cholesterol biosynthesis

**Figure 4.**
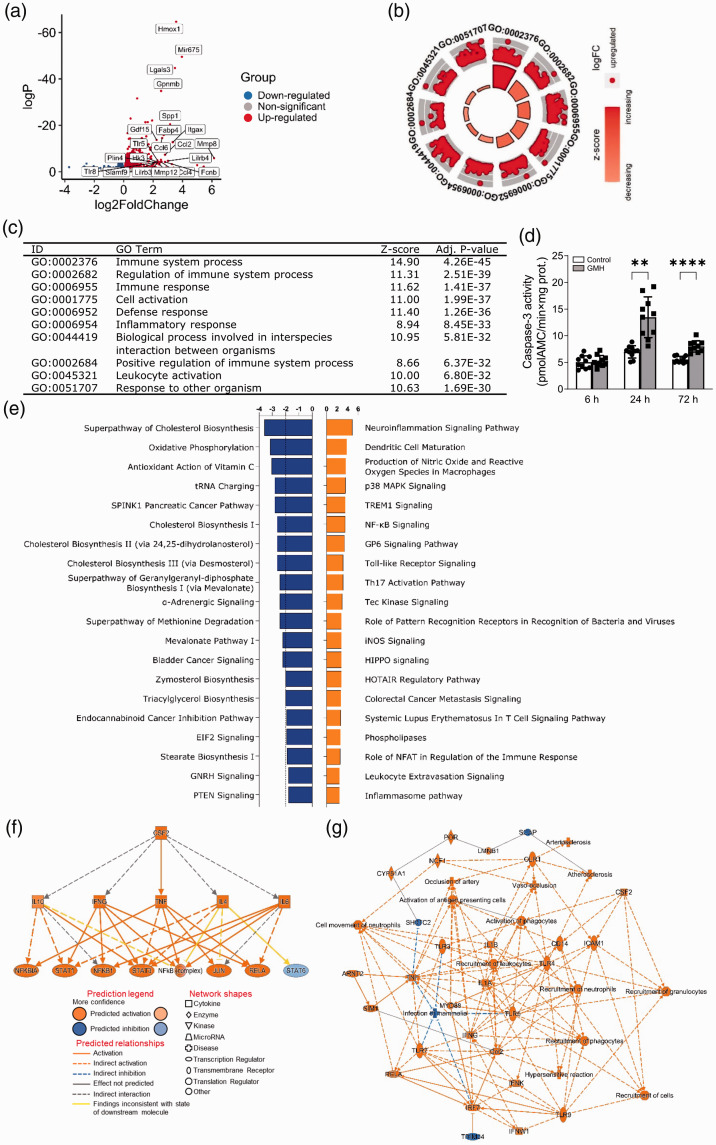
GMH induced inflammation 72 hours after insult. (a) Volcano plot of DEGs at 72 hours following GMH. *P*-adj <0.001 and |(log2FoldChange)| > 1. (b–c) GOplot enrichment analysis of enriched GO biological processes. (d) Caspase-3 activity at 6 hours, 24 hours, and 72 hours following GMH. The canonical pathways (e), regulators (f), and graphical summary of the network (g) predicted by IPA.

There were 1466 DEGs between the control group and the GMH group at 72 hours after GMH induction. The top 20 most significantly upregulated genes are involved in heme catabolism (*Hmox1*), iron metabolism (*Slc11a1*), and immune responses and inflammation (*Lgals3*, *Gpnmb*, *Ctsd*, *Cd68*, *Spp1*, *Slc11a1*, *Grn*, and *Ncf1*). Several of the top upregulated genes also regulate processes related to cell survival, apoptosis, proliferation, and migration (*Mir675*, *Lgals3*, *Gpnmb*, *Megf10*, *Bin2*, *Pdlim4*, *Myo1f*, *Grn,* and *Erbin*) (Figure 4(a), Supplementary data 1, Supplementary Table 5). In addition, several inflammatory chemokines were upregulated, such as *Ccl2*, *Ccl3*, *Ccl4*, *Ccl6*, and *Ccl7* (Supplementary data 1). Genes involved in the mevalonate pathway and cholesterol biosynthesis (*Myd*, *Idi1*, *Tm7sf2*, *Act2*, and *Elovl6*) were among the top 20 most significantly downregulated genes, as well as genes involved in inflammation (*Tbsab1*, *Tbsb2*, and *Agtr2*) and neurodevelopment (*Hes7*, *Nefm*, *Vstm2l*, *Kiss1r*, and *Gjc2*) (Supplementary data 1, Supplementary Table 6).

GOplot analysis revealed upregulation of immunological and inflammatory responses as well as myeloid cell and leukocyte activation (Figure 4(b) and (c), Supplementary data 2 and 3). In addition, neuroinflammation signaling pathways, dendritic cell maturation, and canonical pathways known to regulate inflammation, apoptosis, and cell survival were also predicted to be upregulated by IPA, for example, p38 MAPK signaling, TREM1 signaling, NF-kB signaling, Toll-like receptor signaling, Tec kinase signaling, iNOS signaling, and HIPPO signaling (Figure 4(e), Supplementary data 4). Accordingly, caspase-3 activity was increased at 24 hours and 72 hours following GMH (Figure 4(d)). Furthermore, IPA predicted the mevalonate pathway and cholesterol biosynthesis to be downregulated (Figure 4(e)) and CSF2 to be one of the upstream regulators controlling targets that may be involved in the inflammatory response (Figure 4(f), Supplementary data 5). Recruitment of phagocytes and granulocytes and major innate immune response pathways such as TLR-MyD88 signaling, IL1B, IL1A, and TNF were predicted to be related biological entities (Figure 4(g)).

To further identify the cell types that were potentially involved in the inflammatory response at 72 hours after GMH, we conducted RNA-seq deconvolution analysis using the existing immune cell databases (XCELL and MCPcounter). We found that macrophages and B-cells were the major cell types that are responsible for the immune response at this time point (Supplementary Figure 2 A–B).

### Gene expression changes suggest the occurrence of ferroptosis after GMH

**Figure 5.**
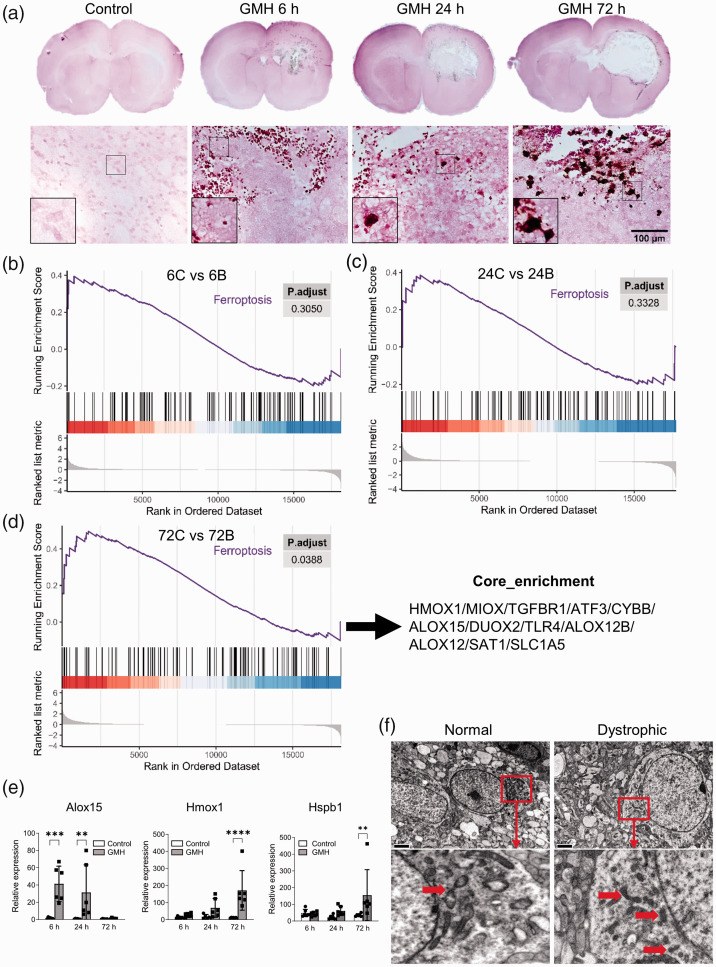
Iron deposition and ferroptosis were observed at multiple time points following GMH. (a) DAB-enhanced Perl's iron staining in the brain at 6 hours, 24 hours, and 72 hours after intracranial injection of collagenase or saline. (b–d) GSEA at 6 hours, 24 hours, and 72 hours after GMH. (e) Analysis of the relative expression levels of *Alox15*, *Hmox1*, and *Hspb1* using reverse transcription qualitative PCR. (f) Normal and dystrophic neurons examined 5 days after GMH using TEM. Arrows indicate mitochondria. Scale bar: 2 µm. C: control, B: GMH.

We have previously shown that iron is heavily accumulated 6 hours post-GMH, while at 24 hours post-GMH the iron in the infarcted area is greatly reduced.^18^ Here we examined this further by also including 72 hours post-GMH. We confirmed that at 6 hours after GMH iron deposition occurred surrounding the hematoma in the peri-hemorrhagic tissue of the striatum (Figure 5(a)). Later, iron accumulation within the core of the injury diminished while the accumulation became more apparent at the border regions around the injured tissue in the striatum, and this was most evident 72 hours after GMH. No iron deposition was observed in the brain of saline-injected control rats (Figure 5(a)).

Ferroptosis is an iron and lipid peroxidation-dependent form of cell death. GSEA showed that genes related to ferroptosis, including *Hmox1*, *Hspb1*, *TGFbr1*, and *ATF3*, were significantly upregulated (Figure 5(b) to (d), Supplementary data 1). In addition, IPA showed that several of the DEGs were predicted to be involved in the ferroptosis pathway at 72 hours following GMH (Supplementary Figure 3). Some DEGs such as *Hmox1* and *Alox15* appeared among the top 20 upregulated genes at different time points (Figures 2 [Fig fig3-0271678X221098811]to 4; Supplementary Table 1 and 3). This was further examined using reverse transcription quantitative PCR. *Alox15* (which is involved in lipid peroxidation and is implicated in ferroptosis^33,34,40^) was significantly increased at 6 hours and 24 hours post-GMH. *Hmox1,* an essential enzyme in heme catabolism that plays an important role in ferroptosis,^40–42^ and *Hspb1* (a regulator of ferroptosis^43^) were significantly increased at 72 hours post-GMH (Figure 5(e)). In addition, TEM analysis at 5 days following GMH showed neurons with shrunken mitochondria in their cytoplasm (Figure 5(f)), which indicates ongoing ferroptosis.^44,45^

### GMH leads to neurodevelopmental deterioration

**Figure 6.**
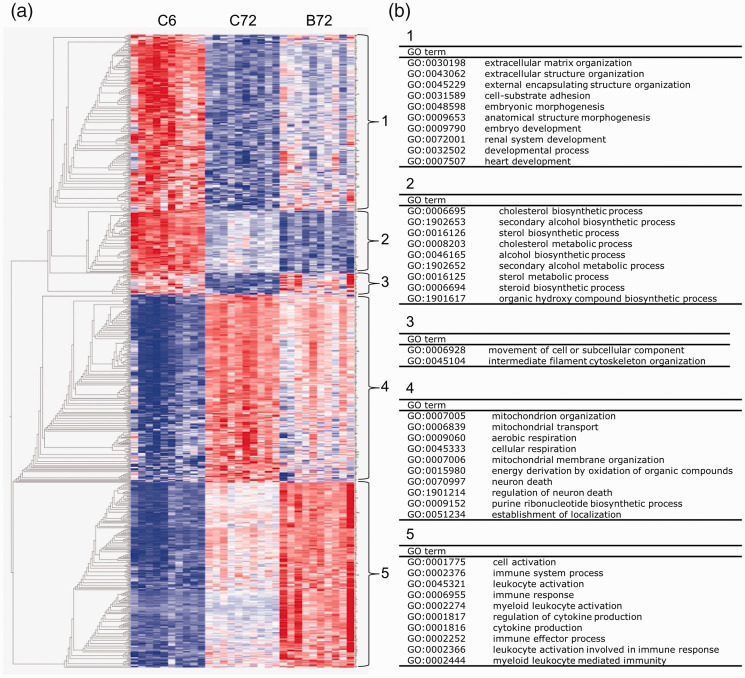
The developmentally regulated processes that showed deviating regulation following GMH. (a) Heatmap of the 719 genes that were differently regulated at 72 hours after GMH compared to the normal neurodevelopmental regulation at P8 *versus* P5 in controls, forming five clusters. (b) The most significant GO biological processes enriched in each cluster. C: control, B: GMH.

Next, we investigated whether GMH affects genes associated with brain development. First, we identified the DEGs between the control group at 72 hours (P8) and the control group at 6 hours post-GMH (P5). Of the top 5000 DEGs (sorted by q-value), there were 719 genes that were differently regulated at 72 hours after GMH. A heatmap with these genes showed five distinct GO clusters with aberrant regulation following GMH (Figure 6(a), Supplementary data 6).

In cluster 1, consisting of genes downregulated at P8 compared to P5 in controls, the genes were downregulated to a lesser degree following GMH than at P8 in controls. GO enrichment analysis demonstrated that these genes were involved in early/embryonic developmental processes (Figure 6(b)). In cluster 2, consisting of genes involved in metabolic pathways such as cholesterol and steroid biosynthesis, the genes were more downregulated following GMH compared to P8 controls. A small cluster of genes involved in intracellular trafficking and cytoskeletal organization (cluster 3) was not downregulated following GMH as it was in P8 controls. In cluster 4, consisting of genes involved in mitochondria function and regulation of neuronal cell death, the genes were upregulated to a lesser degree after GMH compared to P8 controls. As expected, a large cluster (cluster 5) of genes involved in immune responses and inflammation was upregulated after GMH compared to controls at P8.

This analysis suggests that following GMH there is an impairment in neurodevelopment that may be ascribed to the abnormal regulation of embryonic development, including disruption of mitochondrial functions and metabolic pathways that are critical during brain development.

## Discussion

**Figure 7.**
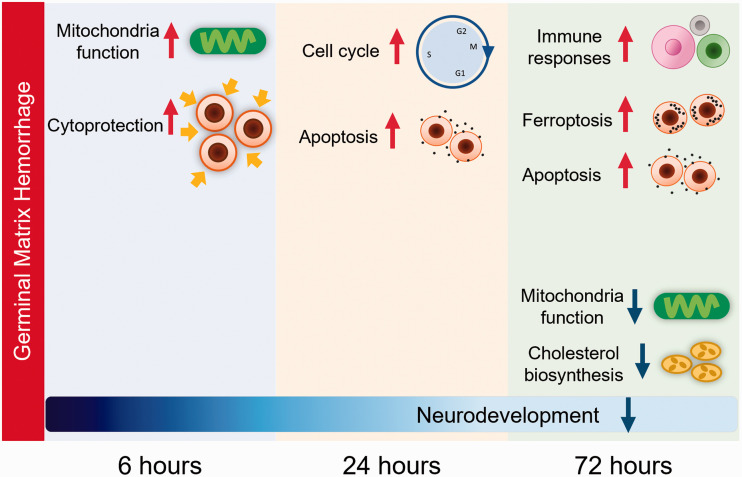
A summary of the major biological events in the brain following induction of GMH in P5 rats.

By performing RNA-seq analysis at different time points following hemorrhage in a rat model of preterm GMH, we have gained novel insights into the temporal changes in the transcriptome profile, signaling pathways, and biological processes that may be significant for the development of brain injury after GMH in the immature brain. Overall, mitochondrial functions were increased at 6 hours after GMH, upregulation of cell cycle progression and downregulation of pathways implicated in neurodevelopment were observed at 24 hours after GMH, and activation of immune responses and inflammation, and ferroptosis were observed at 72 hours after GMH (Figure 7). Further developmental analysis revealed dysregulation of biological processes possibly contributing to neurodevelopmental impairment following GMH.

Mitochondria are the cellular energy factories that produce ATP via oxidative phosphorylation, and they regulate the catabolism of glucose, lipids, and glutamine as well as the production of reactive oxygen species. Mitochondrial function and mitochondria-mediated cytoprotective responses such as protection against oxidative stress through ATP production and heme catabolism are critical during neurodevelopment and in response to neuronal damage.^46,47^ Mitochondrial injury and dysfunction are the main pathological changes and are early events in the pathophysiology of intracerebral hemorrhage,^48–50^ and they trigger the activation of a variety of pathways, including inflammatory responses and cell death pathways.^51^ Indeed, at 6 hours after GMH, the first time point assessed in this study, there was an increase in processes attributable to mitochondrial functions and heme metabolism. In addition, there was upregulation of pathways implicated in cytoprotection. For example, EIF2 signaling was induced, which is of interest because phosphorylation of EIF2 represses global translation and activates genes involved in cytoprotective pathways.^52,53^ The Nrf2 pathway, which was also predicted to be upregulated after GMH, is involved in mitochondrial biogenesis and function^54^ and is cytoprotective by regulating the expression of genes coding for antioxidant, anti-inflammatory, and detoxifying proteins,^55^ and the Nrf2 pathway is also involved in intracerebral hemorrhage in neonatal rats.^56^ For example, Nrf2 induces the expression of *Hmox1* that acts as a cytoprotective factor via its role in heme catabolism,^57^ and *Hmox1* was among the top upregulated genes found in this study. Moreover, Cxcr4 signaling that activates proliferation and survival pathways^58^ was also predicted to be upregulated, as was inositol biosynthesis. Inositol is a key player in several signaling pathways and exerts critical activities in physiological and pathological settings by regulating energy production, cellular metabolism, stress adaptation, cell cycle progression, cell survival, and inflammation.^35,59,60^ Taken together, this suggests that an early response to GMH may be a compensatory biological response, including an increase in mitochondria function, as an attempt to cope with the stress caused by GMH by increasing energy levels and by activating protective and survival pathways.

At 24 hours after GMH there was enrichment in processes promoting cell division, wound healing, inflammatory responses, and myeloid cell homeostasis. This indicates that immune cells such as microglia and macrophages may already start to proliferate around this time point. In contrast, pathways involved in neuronal growth and proliferation were predicted to be downregulated. In addition, there was an enrichment of downregulated pathways important during neurodevelopment e.g., synaptogenesis, Eph/ephrin signaling, AhR signaling, adrenomedullin signaling, and CREB signaling. Furthermore, at this time point inositol metabolism was downregulated, contrary to what was observed at 6 hours after GMH. Eph/ephrin signaling has been implicated in the regulation of processes critical to embryonic development, including axon guidance, formation of tissue boundaries, cell migration, and segmentation.^37^ AhR signaling is involved in neuronal proliferation and differentiation as well as dendritic morphology,^36^ and adrenomedullin is important in multiple aspects of neurodevelopment such as neuron growth and cell fate.^38,61^ CREB is a transcription factor that regulates diverse processes such as synaptic activity and neuronal plasticity,^39^ and inositol is an osmolyte and a precursor for phosphoinositide synthesis, and both phosphoinositides and inositol phosphatases are essential for CNS function.^35^ This demonstrates that already at 24 hours following GMH pathways that are likely to be significant during neurodevelopment were negatively affected.

Microglia are the resident macrophages of the CNS and are activated in response to brain injuries, including preterm brain injuries.^62–64^ GMH induces microglia-mediated inflammatory responses and oxidative stress that damage the tissue surrounding the injury and the white matter.^65–68^ Treatments that reduce inflammation can enhance myelination and neurological recovery after intraventricular hemorrhage.^69^ Multiple inflammatory chemokines and mediators such as *Ccl2*, *Ccl4*, *Ccl6*, *Tlr5*, *Mmp8*, and *Spp1* were upregulated 72 hours after GMH, likely reflecting the activation of microglia. Also, at this time point immune responses and inflammation were the most enriched processes, and pathways involved in the production of inflammatory mediators were predicted to be upregulated. Inflammatory signaling after intracerebral hemorrhage is largely coordinated by NFkB.^70,71^ TREM1 is a key regulator of inflammation, and NFkB is involved in the regulation of microglial TREM1 during neuroinflammation.^72^ Microglial TREM1 has been demonstrated to mediate neuroinflammation via SYK in experimental ischemic stroke in mice^73^ and to modulate microglia polarization via PKCδ/CARD9 signaling after intracerebral hemorrhage in mice.^74^ In the CNS, p38 MAPK is involved in numerous processes such as synaptic function,^75^ and microglial p38 MAPK mediates neuroinflammation.^76^ In this study, signaling pathways mediated by these aforementioned factors and other known inflammatory mediators were predicted to be upregulated, thus confirming the usefulness of these results for further investigations of the mechanisms involved in brain injury development following GMH. We found that the major immune cell types that may be involved were macrophages and B-cells. Accordingly, CSF2, a cytokine secreted by macrophages, was predicted to be one of the upstream regulators possibly involved in the inflammatory response. Macrophages and microglia play a pivotal role in hematoma clearance after GMH,^66,69^ but the pathophysiological role of B-cells after intracerebral hemorrhage has not been well studied. In contrast to our findings, B-cells have been shown to have a low infiltration rate at 1 day and 5 days after insult in an adult mouse model of intracerebral hemorrhage.^77^

The mevalonate pathway synthesizes cholesterol and is also involved in the synthesis of heme, and several genes and pathways regulating the mevalonate pathway and cholesterol biosynthesis were downregulated at 72 hours after GMH. Cholesterol is a major component of organelle and cell membranes, myelin, and steroid precursors, and cholesterol levels in the brain are regulated through its biosynthesis and its trafficking and elimination. Defects in cholesterol biosynthesis and regulation result in neurodevelopmental impairment and in myelination and synaptogenesis anomalies in the CNS.^78^ This suggests that downregulation of cholesterol biosynthesis may contribute to brain injury development following GMH.

Various forms of cell death have been identified after intracerebral hemorrhage, including apoptosis,^79^ necrosis,^80^ and ferroptosis.^45,81^ Consistent with previous reports, our data showed that caspase-3 activity was significantly increased at 24 hours and 72 hours after GMH, indicating apoptotic cell death. Ferroptosis is a recently identified form of cell death that depends on iron and lipid peroxidation.^29,44,82^ Emerging evidence implicates ferroptosis as a mechanism behind several neurological disorders such as ischemic stroke,^83^ neurodegenerative processes,^84^ traumatic brain injury,^85^ and intracerebral hemorrhage.^86^ Administration of ferrostatin-1, a specific inhibitor of ferroptosis, protects the hemorrhagic brain in an animal model.^45^ Our results suggest that ferroptosis is involved in preterm GMH, and the most significantly upregulated genes, i.e., *Hmox1* and *Alox15*, are implicated in ferroptosis and are thus interesting candidates for further investigations into the mechanisms of secondary brain injury.

During neurodevelopment, the differentiation of neurons, astrocytes, and oligodendrocytes is a highly energy demanding process^87^ requiring an increase in mitochondrial biogenesis and function.^88^ By comparing DEGs between P5 controls and P8 controls, we found that there was a developmental enrichment of upregulated biological processes involving mitochondrial functions. However, at 72 hours following GMH this increase was much less obvious. The health and survival of neurons depends largely on the integrity and function of mitochondria, and therefore mitochondrial defects have detrimental effects on the CNS. Hemoglobin and heme released from lysed blood cells are highly reactive and have been shown to disrupt mitochondrial function in different types of injuries,^89,90^ and cell-free hemoglobin following preterm intraventricular hemorrhage has been suggested to be involved in the brain injury process.^68,91–93^ The heme and radical scavenger α1-microglobulin, as well as the iron chelator deferoxamine, have been shown to have neuroprotective effects against brain injury following neonatal intracerebral hemorrhage.^94,95^

We also found that processes enriched during early development, such as extracellular matrix organization and organ development, were not decreased to the same level in brains after GMH compared to normal development. Our data suggest that GMH disrupts the normal developmental regulation of sterol and cholesterol biosynthesis. Metabolism is critical to every aspect of neurodevelopment, and disruption in metabolic processes can produce various clinical manifestations.^96^ Moreover, our data further support the idea that immune responses and inflammatory processes take part in neurodevelopmental deterioration following GMH and provide further insights into the specific genes and pathways that are involved.

Our study has some limitations that should be noted. First of all, the study certainly provides a valuable overview of the sequence of biological events and transcriptome changes following GMH in an animal model, which is currently lacking; however, the findings need biological validation and their relevance to clinical cases of GMH in preterm infants also need more exploration and confirmation using human postmortem brain tissues samples. Of note, there is still a lack of a good animal model that best mimics the clinical features of GMH in preterm infants. There are only a few transgenic models for studying spontaneous GMH, while lesion-induced models are more frequently used, and among them the two most used rodent intracerebral hemorrhage models are blood-induced and collagenase-induced injuries. Both of these models have been demonstrated to have clinical relevance (summarized in^65^) but they also have limitations. Blood administration is technically complicated in newborn rodent pups and does not induce the rupture of vessels, in contrast to the collagenase-induced injury model used here, where only a small volume of collagenase is administered that causes degradation of the basal lamina leading to the rupture of vessels. However, the time course of the collagenase action causing GMH remains unknown.

To our knowledge, this is the first study describing the overall transcriptomic changes and major biological processes underlying GMH pathophysiology in the premature brain in rats. Our main findings highlight pathways involved in early upregulation of mitochondria functions, cytoprotective responses, and heme metabolic processes, followed by subsequent cell death via apoptosis and ferroptosis, immune cell infiltration, and impairment of neurodevelopmental processes. Among these effects, GMH-triggered mitochondrial dysfunction, heme catabolism, downregulation of cholesterol biosynthesis, and inflammation seem to be the key processes critical for the development of secondary brain injury and subsequent neurodevelopmental impairment. Together, the current study offers an important overview and guidance for exploring the molecular mechanisms underlying secondary brain injury following GMH in the immature brain, and such knowledge is critical for the development of effective therapies.

## Supplemental Material

sj-xlsx-1-jcb-10.1177_0271678X221098811 - Supplemental material for Temporal brain transcriptome analysis reveals key pathological events after germinal matrix hemorrhage in neonatal rats Click here for additional data file. Supplemental material, sj-xlsx-1-jcb-10.1177_0271678X221098811 for Temporal brain transcriptome analysis reveals key pathological events after germinal matrix hemorrhage in neonatal rats by Juan Song, Gisela Nilsson, Yiran Xu, Aura Zelco, Eridan Rocha-Ferreira, Yafeng Wang, Xiaoli Zhang, Shan Zhang, Joakim Ek, Henrik Hagberg, Changlian Zhu and Xiaoyang Wang in Journal of Cerebral Blood Flow & Metabolism

sj-xlsx-2-jcb-10.1177_0271678X221098811 - Supplemental material for Temporal brain transcriptome analysis reveals key pathological events after germinal matrix hemorrhage in neonatal rats Click here for additional data file. Supplemental material, sj-xlsx-2-jcb-10.1177_0271678X221098811 for Temporal brain transcriptome analysis reveals key pathological events after germinal matrix hemorrhage in neonatal rats by Juan Song, Gisela Nilsson, Yiran Xu, Aura Zelco, Eridan Rocha-Ferreira, Yafeng Wang, Xiaoli Zhang, Shan Zhang, Joakim Ek, Henrik Hagberg, Changlian Zhu and Xiaoyang Wang in Journal of Cerebral Blood Flow & Metabolism

sj-xlsx-3-jcb-10.1177_0271678X221098811 - Supplemental material for Temporal brain transcriptome analysis reveals key pathological events after germinal matrix hemorrhage in neonatal rats Click here for additional data file. Supplemental material, sj-xlsx-3-jcb-10.1177_0271678X221098811 for Temporal brain transcriptome analysis reveals key pathological events after germinal matrix hemorrhage in neonatal rats by Juan Song, Gisela Nilsson, Yiran Xu, Aura Zelco, Eridan Rocha-Ferreira, Yafeng Wang, Xiaoli Zhang, Shan Zhang, Joakim Ek, Henrik Hagberg, Changlian Zhu and Xiaoyang Wang in Journal of Cerebral Blood Flow & Metabolism

sj-xlsx-4-jcb-10.1177_0271678X221098811 - Supplemental material for Temporal brain transcriptome analysis reveals key pathological events after germinal matrix hemorrhage in neonatal rats Click here for additional data file. Supplemental material, sj-xlsx-4-jcb-10.1177_0271678X221098811 for Temporal brain transcriptome analysis reveals key pathological events after germinal matrix hemorrhage in neonatal rats by Juan Song, Gisela Nilsson, Yiran Xu, Aura Zelco, Eridan Rocha-Ferreira, Yafeng Wang, Xiaoli Zhang, Shan Zhang, Joakim Ek, Henrik Hagberg, Changlian Zhu and Xiaoyang Wang in Journal of Cerebral Blood Flow & Metabolism

sj-xlsx-5-jcb-10.1177_0271678X221098811 - Supplemental material for Temporal brain transcriptome analysis reveals key pathological events after germinal matrix hemorrhage in neonatal rats Click here for additional data file. Supplemental material, sj-xlsx-5-jcb-10.1177_0271678X221098811 for Temporal brain transcriptome analysis reveals key pathological events after germinal matrix hemorrhage in neonatal rats by Juan Song, Gisela Nilsson, Yiran Xu, Aura Zelco, Eridan Rocha-Ferreira, Yafeng Wang, Xiaoli Zhang, Shan Zhang, Joakim Ek, Henrik Hagberg, Changlian Zhu and Xiaoyang Wang in Journal of Cerebral Blood Flow & Metabolism

sj-xlsx-6-jcb-10.1177_0271678X221098811 - Supplemental material for Temporal brain transcriptome analysis reveals key pathological events after germinal matrix hemorrhage in neonatal rats Click here for additional data file. Supplemental material, sj-xlsx-6-jcb-10.1177_0271678X221098811 for Temporal brain transcriptome analysis reveals key pathological events after germinal matrix hemorrhage in neonatal rats by Juan Song, Gisela Nilsson, Yiran Xu, Aura Zelco, Eridan Rocha-Ferreira, Yafeng Wang, Xiaoli Zhang, Shan Zhang, Joakim Ek, Henrik Hagberg, Changlian Zhu and Xiaoyang Wang in Journal of Cerebral Blood Flow & Metabolism

sj-jpg-7-jcb-10.1177_0271678X221098811 - Supplemental material for Temporal brain transcriptome analysis reveals key pathological events after germinal matrix hemorrhage in neonatal rats Click here for additional data file. Supplemental material, sj-jpg-7-jcb-10.1177_0271678X221098811 for Temporal brain transcriptome analysis reveals key pathological events after germinal matrix hemorrhage in neonatal rats by Juan Song, Gisela Nilsson, Yiran Xu, Aura Zelco, Eridan Rocha-Ferreira, Yafeng Wang, Xiaoli Zhang, Shan Zhang, Joakim Ek, Henrik Hagberg, Changlian Zhu and Xiaoyang Wang in Journal of Cerebral Blood Flow & Metabolism

sj-jpg-8-jcb-10.1177_0271678X221098811 - Supplemental material for Temporal brain transcriptome analysis reveals key pathological events after germinal matrix hemorrhage in neonatal rats Click here for additional data file. Supplemental material, sj-jpg-8-jcb-10.1177_0271678X221098811 for Temporal brain transcriptome analysis reveals key pathological events after germinal matrix hemorrhage in neonatal rats by Juan Song, Gisela Nilsson, Yiran Xu, Aura Zelco, Eridan Rocha-Ferreira, Yafeng Wang, Xiaoli Zhang, Shan Zhang, Joakim Ek, Henrik Hagberg, Changlian Zhu and Xiaoyang Wang in Journal of Cerebral Blood Flow & Metabolism

sj-jpg-9-jcb-10.1177_0271678X221098811 - Supplemental material for Temporal brain transcriptome analysis reveals key pathological events after germinal matrix hemorrhage in neonatal rats Click here for additional data file. Supplemental material, sj-jpg-9-jcb-10.1177_0271678X221098811 for Temporal brain transcriptome analysis reveals key pathological events after germinal matrix hemorrhage in neonatal rats by Juan Song, Gisela Nilsson, Yiran Xu, Aura Zelco, Eridan Rocha-Ferreira, Yafeng Wang, Xiaoli Zhang, Shan Zhang, Joakim Ek, Henrik Hagberg, Changlian Zhu and Xiaoyang Wang in Journal of Cerebral Blood Flow & Metabolism

sj-pdf-10-jcb-10.1177_0271678X221098811 - Supplemental material for Temporal brain transcriptome analysis reveals key pathological events after germinal matrix hemorrhage in neonatal rats Click here for additional data file. Supplemental material, sj-pdf-10-jcb-10.1177_0271678X221098811 for Temporal brain transcriptome analysis reveals key pathological events after germinal matrix hemorrhage in neonatal rats by Juan Song, Gisela Nilsson, Yiran Xu, Aura Zelco, Eridan Rocha-Ferreira, Yafeng Wang, Xiaoli Zhang, Shan Zhang, Joakim Ek, Henrik Hagberg, Changlian Zhu and Xiaoyang Wang in Journal of Cerebral Blood Flow & Metabolism

sj-pdf-11-jcb-10.1177_0271678X221098811 - Supplemental material for Temporal brain transcriptome analysis reveals key pathological events after germinal matrix hemorrhage in neonatal rats Click here for additional data file. Supplemental material, sj-pdf-11-jcb-10.1177_0271678X221098811 for Temporal brain transcriptome analysis reveals key pathological events after germinal matrix hemorrhage in neonatal rats by Juan Song, Gisela Nilsson, Yiran Xu, Aura Zelco, Eridan Rocha-Ferreira, Yafeng Wang, Xiaoli Zhang, Shan Zhang, Joakim Ek, Henrik Hagberg, Changlian Zhu and Xiaoyang Wang in Journal of Cerebral Blood Flow & Metabolism
