# Supplementary figures and images for: Temporal brain transcriptome analysis reveals key pathological events after germinal matrix hemorrhage in neonatal rats
Source: J Cereb Blood Flow Metab. 2022 May 1;42(9):1632–49. doi: 10.1177/0271678X221098811 (PMC9441725; doi:10.1177/0271678X221098811)

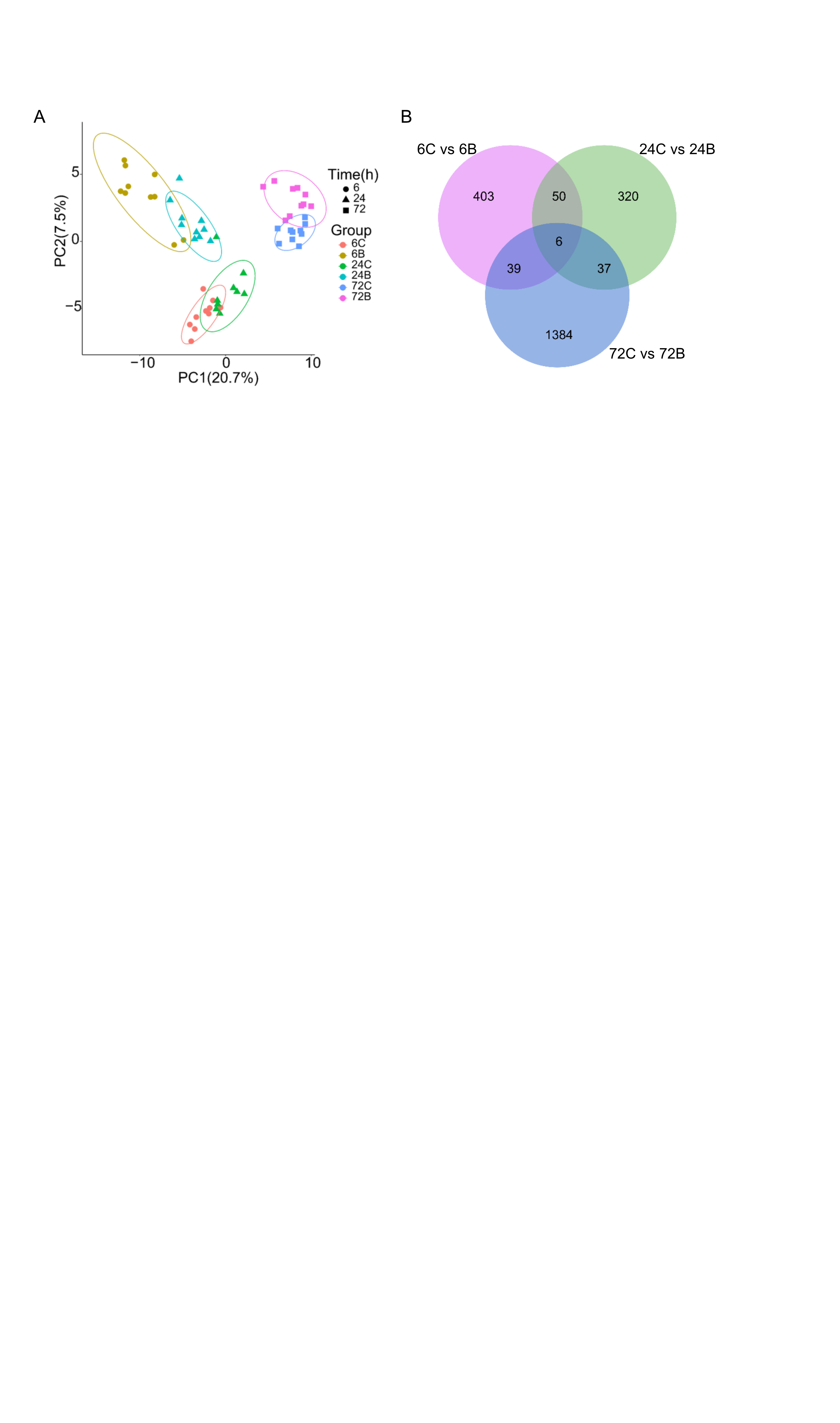

Supplement: sj-jpg-7-jcb-10.1177_0271678X221098811 - Supplemental material for Temporal brain transcriptome analysis reveals key pathological events after germinal matrix hemorrhage in neonatal rats [file sj-jpg-7-jcb-10.1177_0271678X221098811.jpg]

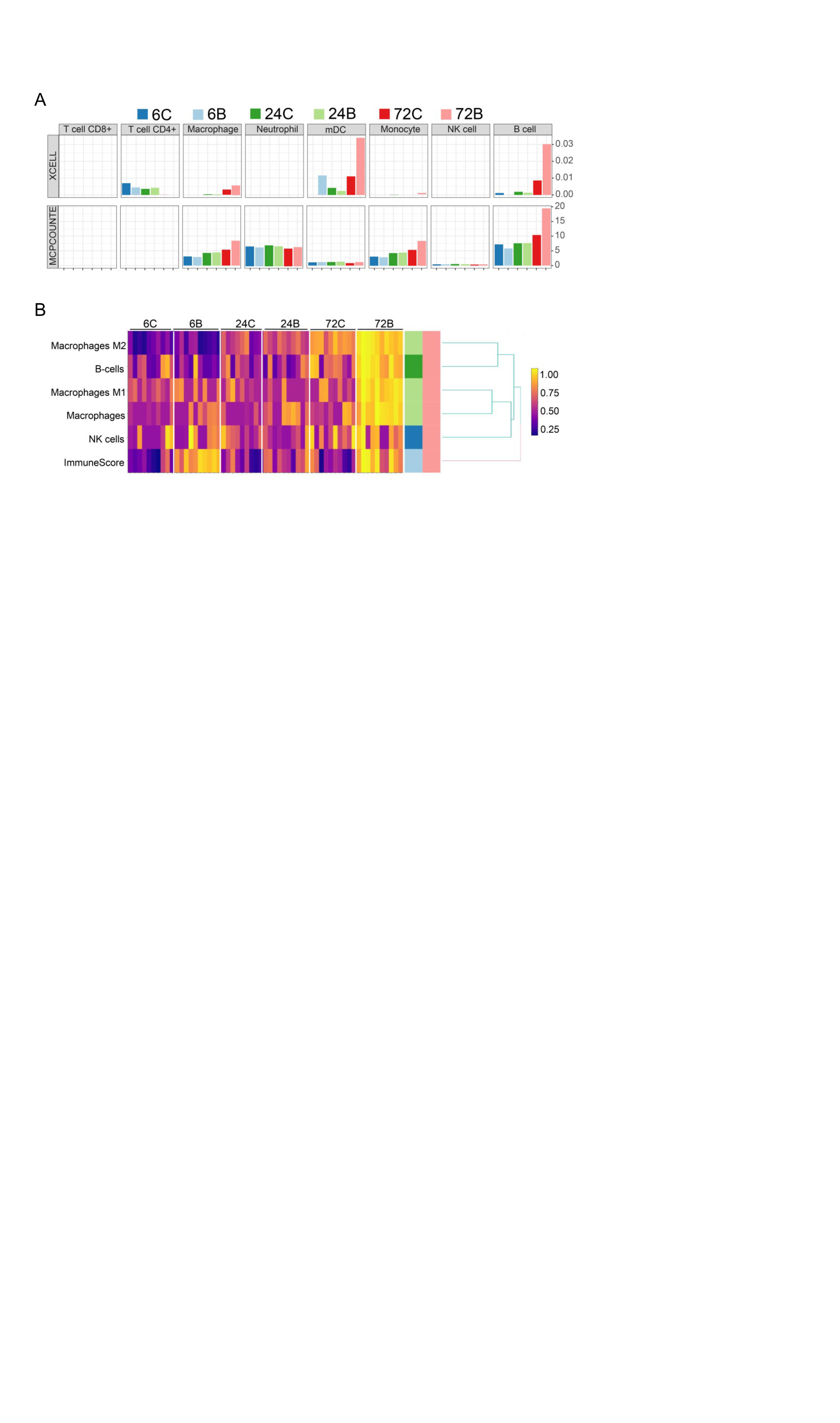

Supplement: sj-jpg-8-jcb-10.1177_0271678X221098811 - Supplemental material for Temporal brain transcriptome analysis reveals key pathological events after germinal matrix hemorrhage in neonatal rats [file sj-jpg-8-jcb-10.1177_0271678X221098811.jpg]

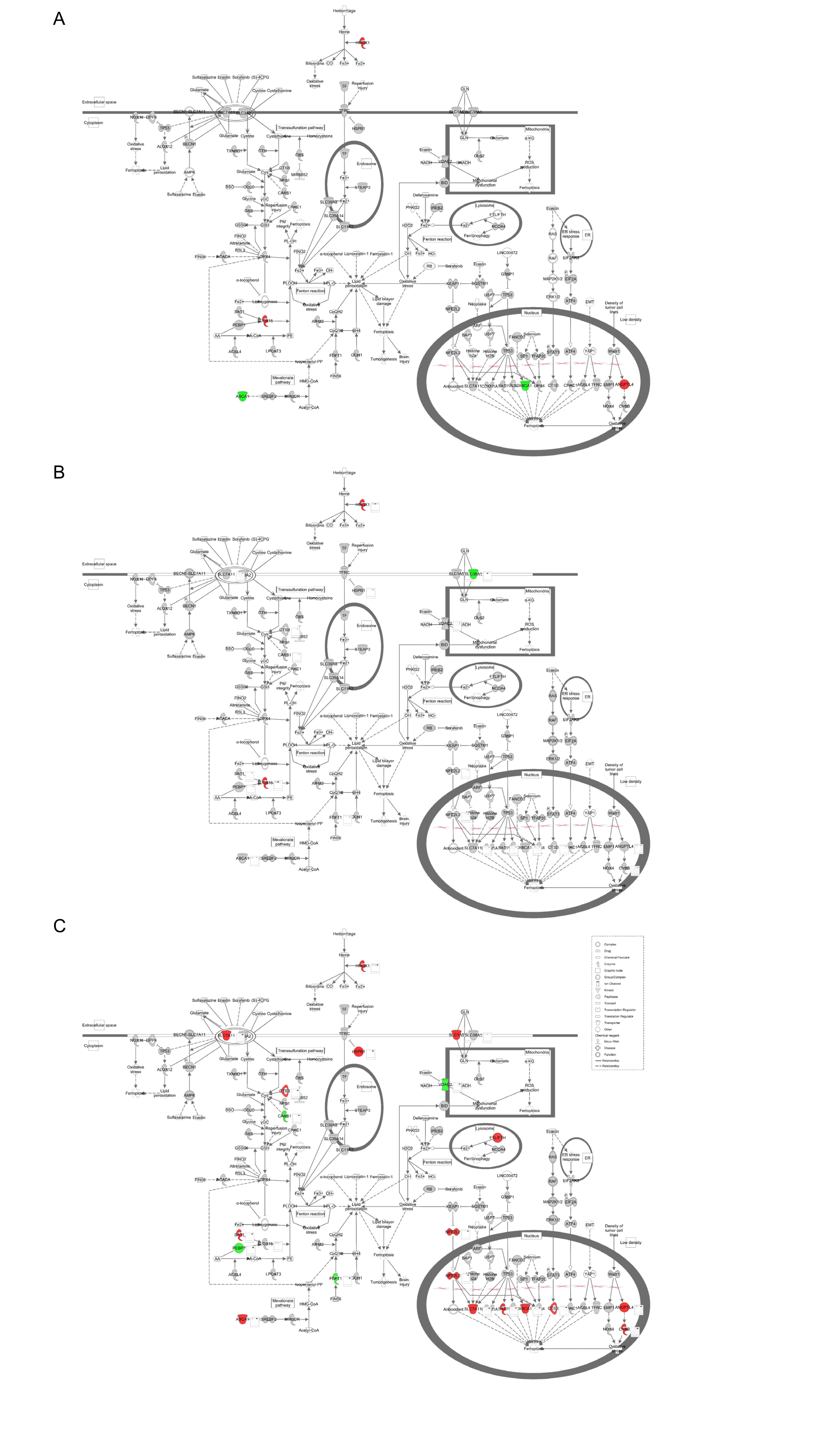

Supplement: sj-jpg-9-jcb-10.1177_0271678X221098811 - Supplemental material for Temporal brain transcriptome analysis reveals key pathological events after germinal matrix hemorrhage in neonatal rats [file sj-jpg-9-jcb-10.1177_0271678X221098811.jpg]
