# Supplementary material for: Temporal brain transcriptome analysis reveals key pathological events after germinal matrix hemorrhage in neonatal rats
Source: J Cereb Blood Flow Metab. 2022 May 1;42(9):1632–49. doi: 10.1177/0271678X221098811 (PMC9441725; doi:10.1177/0271678X221098811)
Supplement: sj-pdf-10-jcb-10.1177_0271678X221098811 - Supplemental material for Temporal brain transcriptome analysis reveals key pathological events after germinal matrix hemorrhage in neonatal rats [file sj-pdf-10-jcb-10.1177_0271678X221098811.pdf]

Table S1. Top 20 upregulated DEGs in GMH *versus* control at 6 hours after GMH.

Gene	Gene description	FC	P-value adj	Function
Alox15	Arachidonate 15-lipoxygenase	26.8	1.1E-38	Polyunsaturated fatty acid metabolism, inflammation, ferroptosis
Hba-a2	Hemoglobin alpha, adult chain 2	37.6	3.6E-38	Hemoglobin component
Alas2	Delta-aminolevulinate synthase 2	34.1	4.6E-33	Heme biosynthesis
Hbb	Hemoglobin beta chain complex	46.0	5.5E-30	Hemoglobin component
Hba-a1	Hemoglobin alpha, adult chain 1	36.1	3.2E-28	Hemoglobin component
Ybx3	Y box protein 3	1.7	1.1E-23	DNA/RNA-binding, cell survival, proliferation, differentiation
Slc25a39	Solute carrier family 25. member 39	1.4	1.1E-14	Amino acid transport, iron homeostasis
H4c8	H4 clustered histone 8	1.7	4.1E-14	Core component of nucleosome
Car2	Carbonic anhydrase 2	1.4	2.5E-13	Hydration of carbon dioxide, heme biosynthesis, energy metabolism
Cdc25b	Cell division cycle 25B	1.4	3.0E-13	Cell cycle progression
Hmox1	Heme oxygenase 1	3.1	1.1E-12	Heme catabolism, ferroptosis
Gadd45g	Growth arrest and DNA-damage-inducible 45 gamma	1.5	1.3E-12	Cell cycle control, DNA repair, apoptosis
Rbm38	RNA binding motif protein 38	1.9	1.0E-09	Cell cycle control, mRNA stability
Junb	Jun B proto-oncogene	1.3	8.6E-09	Transcription factor, cell cycle control, cell survival
F3	Coagulation factor III	1.4	1.6E-08	Initiate blood coagulation
Hba-a3	Hemoglobin alpha, adult chain 3	47.4	1.1E-07	Hemoglobin component
Fech	Ferrochelatase	1.2	1.1E-07	Heme biosynthesis
Apln	Apelin	1.4	5.7E-06	Homeostatic regulation of body fluid
Egr1	Early growth response protein 1	1.4	1.1E-05	Transcription factor, cell cycle control, differentiation
Ucp2	Mitochondrial uncoupling protein 2	1.4	4.7E-05	Uncoupling of oxidative phosphorylation

Fold change (FC) > 1.2. Sorted on adjusted p-value.

Table S2. Top 20 downregulated DEGs in GMH *versus* control at 6 hours after GMH.

Gene	Gene description	FC	p-value adj	Function
Klhl34	Kelch-like family member 34	-1.2	2.4E-04	Unknown
Stk35	Serine/threonine kinase 35	-1.2	7.7E-04	Cell cycle control, cell migration
Susd5	Sushi domain containing 5	-1.4	1.1E-03	Unknown
Smcr8	Smith-Magenis syndrome chromosome region candidate 8	-1.2	6.5E-03	Autophagy, guanine nucleotide exchange factor activity
Kcnh7	Potassium voltage-gated channel member 7	-1.3	6.5E-03	Membrane potential regulation
Fat3	FAT atypical cadherin 3	-1.3	7.4E-03	Neuronal morphology, interaction between neurites
Bach2	BTB domain and CNC Homolog 2	-1.3	7.6E-03	Immunity, apoptosis, negative regulator of Hmox1
Abca1	ATP-binding cassette sub-family A member 1	-1.2	1.7E-02	Cholesterol and phospholipid homeostasis
Shc3	Src homology 2 domain containing transforming protein 3	-1.2	1.8E-02	Neuronal signaling
Fras1	Fraser extracellular matrix complex subunit 1	-1.2	1.9E-02	Epidermal-basement membrane adhesion, organogenesis
Klf12	Krueppel-like factor 12	-1.2	2.7E-02	Neural tube development, negatively regulates Enolas 2
Irs1	Insulin receptor substrate 1	-1.2	2.8E-02	Cell cycle progression, metabolism
Rorb	RAR related orphan receptor B	-1.2	3.1E-02	Organogenesis, differentiation
Tenm4	Teneurin transmembrane protein 4	-1.2	3.2E-02	Neuronal connectivity, axon guidance, oligodendrocyte development
Kcna3	Potassium voltage-gated channel subfamily A member 3	-1.2	3.4E-02	Neurotransmitter release, microglial activation, T-cell activation
Thsd7a	Thrombospondin type 1 domain containing 7A	-1.3	3.7E-02	Cytoskeletal organization, cell migration, angiogenesis
Klhl11	Kelch-like family member 11	-1.2	3.7E-02	Predicted role in T-cell response
Dab2	Disabled homolog 2	-1.2	3.7E-02	Clathrin-mediated endocytosis, cell signaling, proliferation, differentiation
Igsf9b	Immunoglobulin Superfamily Member 9B	-1.3	4.1E-02	Inhibitory synapse development
Ino80d	Ino08 complex subunit D	-1.3	4.6E-02	Predicted role in transcriptional regulation, DNA replication and repair

Fold change (FC) < -1.2. Sorted on adjusted p-value.

Table S3. Top 20 upregulated DEGs in GMH *versus* control at 24 hours after GMH.

Gene	Gene description	FC	p-value adj	Function
Hmox1	Heme oxygenase 1	4.9	9.73E-16	Heme catabolism, ferroptosis
Lgals5	Galectin 5	12.1	2.18E-11	Predicted role erythropoiesis, exosomal sorting
Hba-a1	Hemoglobin alpha, adult chain 1	9.6	7.21E-11	Hemoglobin component
Hbb	Hemoglobin beta chain complex	9.9	8.45E-11	Hemoglobin component
Hba-a2	Hemoglobin alpha, adult chain 2	10.0	5.57E-10	Hemoglobin component
Timp1	TIMP metallopeptidase inhibitor 1	1.6	1.20E-08	Proliferation, cell cycle control, cell death, erythropoiesis
Alas2	Delta-aminolevulinate synthase 2	6.6	4.77E-07	Heme biosynthesis
Mt2A	Metallothionein 2A	1.8	4.77E-07	Antioxidant, heavy metal toxicity protection
Alox15	Arachidonate 15-lipoxygenase	4.7	4.31E-06	Polyunsaturated fatty acid metabolism, inflammation, ferroptosis
Cdk1	Cyclin-dependent kinase 1	1.2	4.31E-06	Cell cycle progression
Tubb6	Tubulin beta 6 chain	1.4	4.72E-06	Cell cycle, microtubule component, mitotic spindle component
Ifitm3	Interferon-induced transmembrane protein 3	1.6	2.01E-05	Immune response, disrupt cholesterol homeostasis
Pttg1	PTTG1 regulator of sister chromatid separation, Securin	1.3	2.17E-05	Control of the metaphase-anaphase transition
Denn2b	DENN domain containing 2B	1.3	3.05E-05	Cytoskeletal organization, cell migration
Fcgr3a	Low affinity immunoglobulin gamma Fc region receptor III-A	1.8	7.40E-05	Antibody-dependent natural killer cell-mediated cytotoxicity
Bin2	Bridging integrator 2	1.4	1.36E-04	Cell motility and migration
Ccnb1	Cyclin B1	1.2	1.36E-04	Cell cycle progression
Arhgap11a	Rho GTPase-activating protein 11A	1.2	3.87E-04	Cell cycle-dependent motility
Cd63	CD63 molecule	1.2	1.35E-03	Cell growth and motility, marker for platelet activation
Cdca8	Cell division cycle associated 8	1.2	1.46E-03	Cell cycle, microtubule stabilization and spindle formation

Fold change (FC) > 1.2. Sorted on adjusted p-value.

Table S4. Top 20 downregulated DEGs in GMH *versus* control at 24 hours after GMH.

Gene	Gene description	FC	p-value adj	Function
Hcfc1	Host cell factor 1	-1.3	3.0E-05	Cell cycle control
Clstn2	Calsyntenin 2	-1.2	1.5E-04	Predicted role in postsynaptic signaling
Ndst1	N-Deacetylase and N-sulfotransferase 1	-1.2	8.2E-04	Heparan sulfate biosynthesis, inflammation
Prrc2b	Proline rich coiled-coil 2B	-1.2	8.3E-04	Predicted to have a role in cell differentiation and neurodevelopment
Ttbk2	Tau tubulin kinase 2	-1.2	1.9E-03	Microtubule stabilization in neurons, cell cycle control, ciliogenesis
Mecp2	Methyl-CpG binding protein 2	-1.3	1.9E-03	Chromatin structure, neurodevelopment
Hip1r	Huntingtin-interacting protein 1-related protein	-1.2	3.8E-03	Intracellular vesicle trafficking, dendritic growth and branching
Pitpnm2	Phosphatidylinositol transfer protein membrane associated 2	-1.3	5.1E-03	Phospholipid biosynthesis and metabolism
Fat3	FAT atypical cadherin 3	-1.2	5.3E-03	Neuronal morphology, interaction between neurites
Tenm4	Teneurin transmembrane protein 4	-1.2	7.6E-03	Neuronal connectivity, axon guidance, oligodendrocyte development
Abcb9	ATP binding cassette subfamily B member 9	-1.2	8.0E-03	Peptide transport, peptide degradation, antigen processing
Mib1	MIB E3 ubiquitin protein ligase 1	-1.3	1.0E-02	Ubiquitination, apoptosis, cell cycle control
Fbxl12	F-box and leucine rich repeat protein 12	-1.2	1.1E-02	Ubiquitination, cell cycle control
Fbxl18	F-box and leucine rich repeat protein 18	-1.2	1.5E-02	Ubiquitination, cell cycle control
Sv2c	Synaptic vesicle glycoprotein 2C	-1.3	1.8E-02	Neurotransmission, vesicle fusion
Tnr	Tenascin R	-1.2	1.9E-02	Neurite outgrowth, cell adhesion, differentiation, sodium channel function
Egr4	Early growth response protein 4	-1.2	2.1E-02	Mitogenesis, differentiation
Fbxo32	F-box protein 32	-1.3	2.3E-02	Ubiquitination, cell cycle control, apoptosis
Myo5a	Myosin VA	-1.2	2.4E-02	Vesicle transport, action potential, spindle pole formation
Ksr2	Kinase suppressor of Ras 2	-1.3	2.4E-02	Fatty acid oxidation, cell growth, energy- and glucose homeostasis

Fold change (FC) < -1.2. Sorted on adjusted p-value.

Table S5. Top 20 upregulated DEGs in GMH *versus* control at 72 hours after GMH.

Gene	Gene description	FC	p-value adj	Function
Hmox1	Heme oxygenase 1	11.8	2.4E-65	Heme catabolism, ferroptosis
Mir675	microRNA 675	15.5	3.1E-50	Proliferation, apoptosis, cell migration
Lgals3	Galectin 3	11.2	2.1E-45	Inflammation, apoptosis, cell adhesion, migration
Gpnmb	Glycoprotein Nmb	5.8	1.5E-35	Inflammation, proliferation, differentiation
Abca1	ATP-Binding cassette sub-family A member 1	1.9	2.5E-32	Cholesterol and phospholipid homeostasis
Ctsd	Cathepsin D	1.4	1.1E-23	Lysosomal recycling of substrates, apoptosis, autophagy, inflammation
Plau	Plasminogen activator, Urokinase	3.8	8.0E-23	Converts plasminogen into plasmin, promotes dissolution of blood coagulates
Cd68	CD68 molecule	3.1	4.1E-22	Lysosomal metabolism, phagocytosis, inflammation
Pla2g7	Phospholipase A2 group VII	2.8	4.5E-22	Degradation of platelet-activating factor
Spp1	Secreted phosphoprotein 1, Osteopontin	8.9	3.5E-21	Immune response, chemokine, cell activation, apoptosis
Megf10	Multiple epidermal growth factor-like domains protein 10	1.5	1.3E-20	Cell adhesion, motility, proliferation, apoptotic cell phagocytosis
Slc11a1	Solute carrier family 11 member 1	2.9	4.3E-19	Iron metabolism, inflammation
Bin2	Bridging integrator 2	1.8	4.2E-18	Cell motility and migration
Pdlim4	PDZ and LIM domain 4	1.6	7.6E-18	Cell migration
Cfh	Complement factor H	1.9	1.8E-17	Regulation of complement activation
Myo1f	Myosin 1F	2.3	2.3E-17	Intracellular movements, actin cytoskeletal dynamics, cell migration
Grn	Granulin precursor	1.4	2.4E-17	Cell growth, cell survival, proliferation, inflammation
Erbin	Erbb2 interacting protein	1.2	2.4E-17	Cell growth, cell survival, proliferation
Fabp4	Fatty acid binding protein 4	6.6	1.5E-16	Fatty acid transport and metabolism
Ncf1	Neutrophil cytosolic factor 1	2.9	5.4E-16	Superoxide production, inflammation

Fold change (FC) > 1.2. Sorted on adjusted p-value.

Table S6. Top 20 downregulated DEGs in GMH *versus* control at 72 hours after GMH.

Gene	Gene description	FC	p-value adj	Function
Klhl32	Kelch like family member 32	-1.2	8.3E-06	Unknown
Mvd	Mevalonate diphosphate decarboxylase	-1.2	1.9E-05	Mevalonate pathway, cholesterol biosynthesis
Alas1	5'-Aminolevulinate synthase 1	-1.2	2.2E-05	Heme biosynthesis
Idi1	Isopentenyl-diphosphate delta isomerase 1	-1.2	3.4E-05	Mevalonate pathway, cholesterol biosynthesis
Tm7sf2	Transmembrane 7 superfamily member 2	-1.2	3.5E-05	Mevalonate pathway, cholesterol biosynthesis
Rbmxl1b	RNA-binding motif protein, X-linked-like 1B	-1.2	1.8E-04	Unknown
Hes7	Hes family BHLH transcription factor 7	-1.2	1.1E-03	Notch-signaling, somitogenesis, neurodevelopment
Qpct	Glutaminyl-peptide cyclotransferase	-1.2	2.6E-03	Cyclic glutamyl peptide biosynthesis
Nefm	Neurofilament medium chain	-1.2	3.0E-03	Component of the axoskeleton
Tpsab1	Tryptase alpha/beta 1	-2.5	3.6E-03	Inflammation, expressed by mast cells, extracellular matrix degradation
Tpsb2	Tryptase beta 2	-2.1	3.6E-03	Inflammation, expressed by mast cells, extracellular matrix degradation
Rtl3	Retrotransposon Gag like 3	-4.1	4.1E-03	Retrotransposon-derived transcript
Vstm2l	V-set and transmembrane domain containing 2 like	-1.2	4.7E-03	Cell adhesion, neuronal viability
Casp6	Caspase 6	-6.4	4.7E-03	Apoptosis, immune response
Prkch	Protein kinase C eta	-1.2	6.3E-03	Cell proliferation, differentiation, cell death, activated by cholesterol
Kiss1r	KISS1 receptor	-1.2	6.5E-03	Neuroendocrine regulation, sensing of metabolic cues
Gjc2	Gap junction gamma 2	-1.4	8.1E-03	Myelination, cell adhesion
Acat2	Acetyl-CoA acetyltransferase 2	-1.2	8.5E-03	Mevalonate pathway, cholesterol biosynthesis
Elovl6	ELOVL fatty acid elongase 6	-1.9	1.1E-02	Mevalonate pathway, cholesterol biosynthesis
Agtr2	Angiotensin II receptor type 2	-1.6	1.3E-02	Apoptosis, inflammation

Fold change (FC) < -1.2. Sorted on adjusted p-value.
