# Supplementary material for: Temporal brain transcriptome analysis reveals key pathological events after germinal matrix hemorrhage in neonatal rats
Source: J Cereb Blood Flow Metab. 2022 May 1;42(9):1632–49. doi: 10.1177/0271678X221098811 (PMC9441725; doi:10.1177/0271678X221098811)
Supplement: sj-pdf-11-jcb-10.1177_0271678X221098811 - Supplemental material for Temporal brain transcriptome analysis reveals key pathological events after germinal matrix hemorrhage in neonatal rats [file sj-pdf-11-jcb-10.1177_0271678X221098811.pdf]

Temporal brain transcriptome analysis reveals key pathological events after germinal matrix hemorrhage in neonatal rats

Running title: Germinal matrix hemorrhage transcriptome changes

Juan Song[1,2*], Gisela Nilsson[1*], Yiran Xu[2*], Aura Zelco[1], Eridan Rocha-Ferreira[3], Yafeng Wang[4,5], Xiaoli Zhang[2], Shan Zhang[2,5], Joakim Ek[1], Henrik Hagberg[3], Changlian Zhu[2,5#], Xiaoyang Wang[1,2,3#]

[1]Centre for Perinatal Medicine and Health, Institute of Neuroscience and Physiology, Sahlgrenska Academy, University of Gothenburg, Gothenburg, Sweden

[2]Henan Key Laboratory of Child Brain Injury and Henan Pediatric Clinical Research Center, Third Affiliated Hospital, and Institute of Neuroscience, of Zhengzhou University, Zhengzhou 450052, China

[3]Centre for Perinatal Medicine and Health, Institute of Clinical Sciences, University of Gothenburg, Gothenburg, Sweden

[4]Henan Provincial Key Laboratory of Children's Genetics and Metabolic Diseases, Children's Hospital Affiliated to Zhengzhou University, Zhengzhou, 450018, China

[5]Center for Brain Repair and Rehabilitation, Institute of Neuroscience and Physiology, Sahlgrenska Academy, University of Gothenburg, Sweden

*Equal contribution first author

#Equal contribution senior author

Corresponding authors:

Xiaoyang Wang, MD, PhD, Professor, Centre for Perinatal Medicine and Health, Institute of Clinical Sciences, University of Gothenburg. Postal address: Box 432, SE-405 30 Gothenburg, Sweden, Tel: +46 31 786 3260; Email: xiaoyang.wang@fysiologi.gu.se

Gisela Nilsson, PhD, Department of Physiology, Institute of Neuroscience and Physiology, University of Gothenburg. Postal address: Box 432, SE-405 30 Gothenburg, Sweden, Tel: +46 737 308145; Email: gisela.nilsson@neuro.gu.se

Supplemental material

Supplementary figure 1.

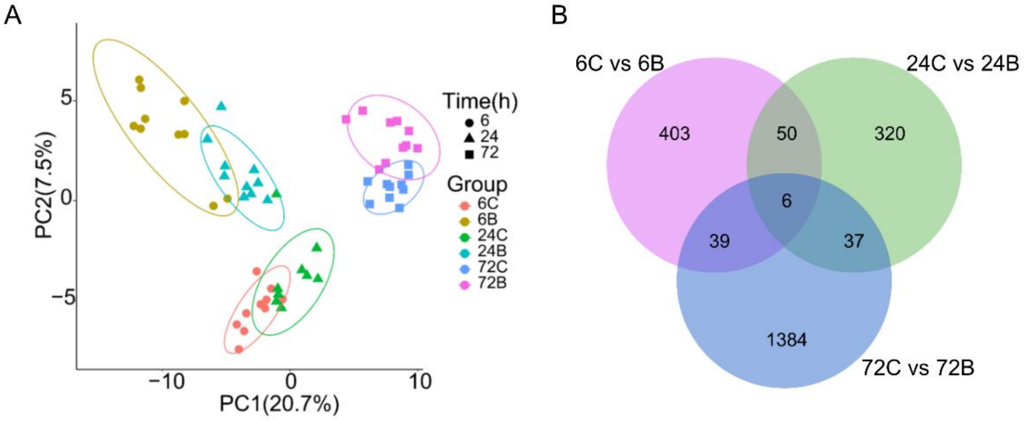

Supplementary figure 1. Differential gene expression after GMH. RNA-seq was performed on brain samples from control and GMH groups at 6 hours, 24 hours, and 72 hours after induction of GMH. (A) PCA plot showing the separation of the different groups with regard to treatment as well as time point. (B) Venn diagram of significant DEGs in control *versus* GMH groups at the different time points. All DEGs had an adjusted *p*-value < 0.05. C: control, B: GMH.

Supplementary figure 2.

A

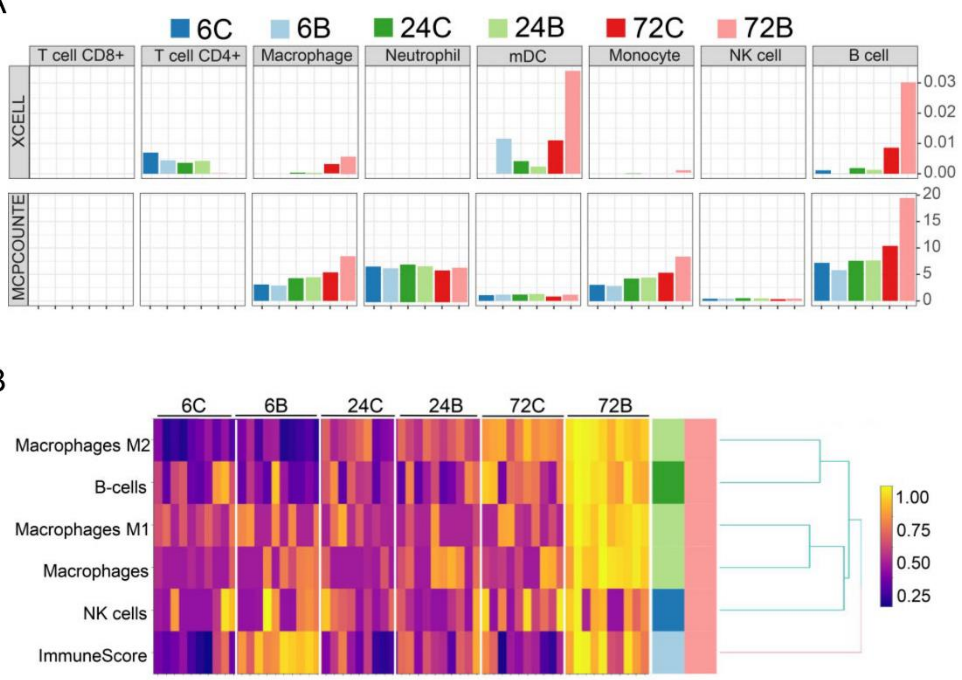

Supplementary figure 2. GMH induced immune cell infiltration at 72 hours after insult. The proportion of different types of immune cells in the brain after GMH shown as a histogram in (A) and as a heatmap in (B) based on the RNA-seq deconvolution algorithm method using the immune cell databases XCELL and MCPCOUNTE. C: control, B: GMH.

Supplementary figure 3.

A

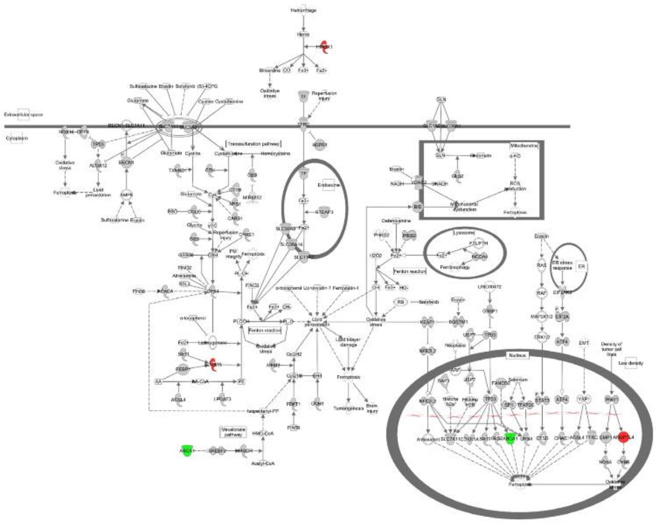

B

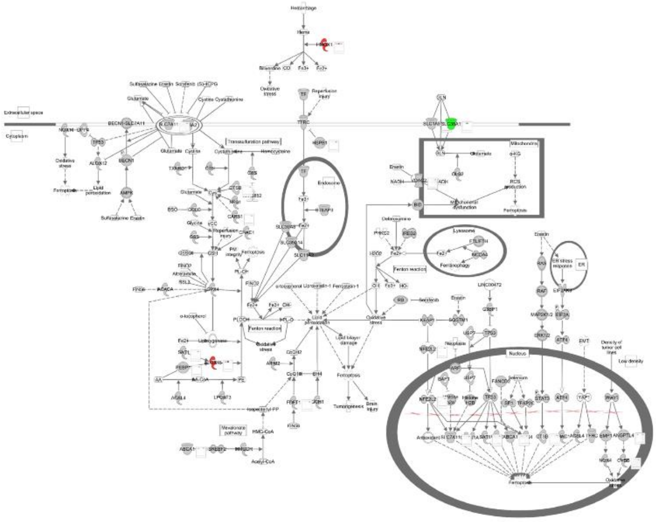

C

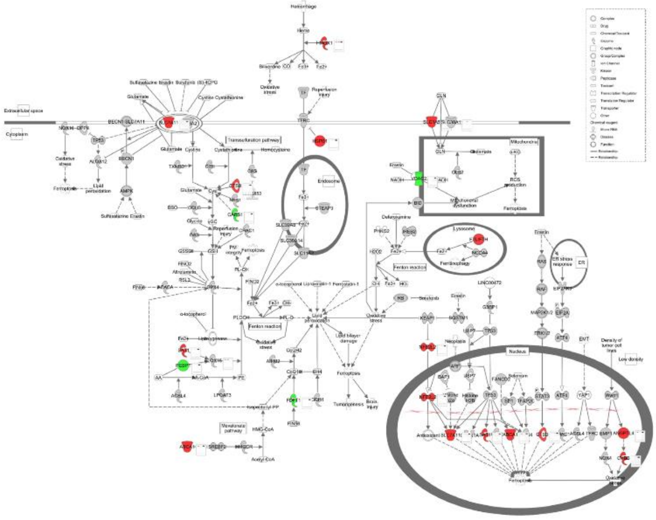

Supplementary figure 3. IPA predicted the involvement of multiple DEGs in the

ferroptosis signaling pathway at 72 hours after GMH. The ferroptosis pathway was analyzed using IPA at 6 hours (A), 24 hours (B), and 72 hours (C) after GMH. Genes with red color: up-regulation, and green color: down-regulation.
